# Multiple Sclerosis and Pregnancy: Current Considerations

**DOI:** 10.1155/2014/513160

**Published:** 2014-04-07

**Authors:** Ioan Buraga, Roxana-Elena Popovici

**Affiliations:** ^1^Head of Neurology, “Carol Davila” University of Medicine and Pharmacy, Colentina Clinical Hospital, No. 19-21 Stefan cel Mare Avenue, 020125 Bucharest, Romania; ^2^Department of Neurology, Colentina Clinical Hospital, No. 19-21 Stefan cel Mare Avenue, 020125 Bucharest, Romania

## Abstract

Multiple sclerosis is the most common neurological disease of young adults that causes major disability. In Romania, it is estimated that this disease has a prevalence of 35–40 per 100,000 inhabitants. It is a disease that begins at the age of 20–40 years and is 2-3 times more common in women than in men. More than half of patients with MS develop the disease in their fertile period of life; therefore, MS patients use contraceptive methods while being under our treatment. Since several therapeutic options have been implemented with good efficiency in the disease stabilization, increasingly more patients begin to wonder about the possibility of having a child and about the possible risks of pregnancy. The evolution during pregnancy and the lactation period has been favorable, with lower relapses and side effects comparable to those in the general population. In addition, babies born to mothers with MS have not had a significantly different mean gestational age or birth weight compared to babies born to healthy mothers.

## 1. Introduction

MS is the most common neurological disease of young adults that causes major disability. In Romania, it is estimated that this disease has a prevalence of 35–40 per 100,000 inhabitants. It is a disease that begins at the age of 20–40 years and is 2-3 times more common in women than in men. More than half of patients with MS develop the disease in the fertile period of life while being under treatment, and, therefore, they have to use contraceptive methods to avoid pregnancy.

Before 1950, most women with MS were counseled to avoid pregnancy because it was thought the disease could be worsened. Over the past 40 years, many studies have been done on hundreds of women with MS, and they have almost all reached the opposite conclusion that pregnancy reduces the number of MS exacerbations, especially in the third trimester.

In a large prospective study of 254 pregnant women with multiple sclerosis, the rate of relapse was 0.7 ± 0.9 per woman per year in the year before pregnancy, 0.5 ± 1.3 during the first trimester, 0.6 ± 1.6 during the second trimester, and 0.2 ± 1.0 during the third. This statistic shows that the frequency of relapses has decreased during pregnancy, especially during the third trimester, but the same study shows an increased rate of 1.2 ± 2.0 during the first three months postpartum which then returned to the prepregnancy rate [1].

More than half of patients with MS develop the disease in the fertile period of life during treatment, and they have to use various contraceptives. The effectiveness of disease-modifying therapy has made more and more patients want their first child, in view of a significant decrease in the annual relapse rate and periods of stable disease.

There have been several cases of patients with MS who were registering a stabilized trend, from both clinical and imaging point of view, when approaching the age of 30 and, therefore, willing for a scheduled pregnancy to be developed in optimal conditions without adverse effects on the mother or newborn.

The current advice is to discontinue disease-modifying treatment (DMT) prior to conception, although studies, which have considered this aspect, have found only minor adverse effects of interferons (IFN) and no effect of glatiramer acetate (GA) [2, 3].

## 2. Our Clinical Experience

Colentina Clinical Hospital is one of the largest centers of MS in the country. At the moment, we have a total of 427 patients under the treatment program with interferons (Avonex, Rebif, and Betaferon) or GA. Of all patients, 129 are men and 298 are women and 68.7% (205 patients) are at fertility age (18–40 years).

Our recommendation supports early initiation of immunomodulatory therapy in order to achieve stabilization of the disease. This stabilization occurs after a minimum of 3 years and a maximum of 5 years. During this period, some patients have given up the desire to have children because of the disease. Lately, increasingly more patients begin to wonder about the possibility of having a child and about the possible risks.

We have 21 patients that have interrupted immunomodulatory treatment when they made the decision to have a child and 18 patients who have discontinued the treatment in the first trimester of pregnancy.

We have a total of 34 healthy children, including one twin pregnancy; one birth defect (foramen ovale); one stillbirth and 3 spontaneous abortions; mothers whose babies were born healthy were exposed to interferon therapy as well as to GA. Birth defects occurred in a patient who discontinued the medication before becoming pregnant, stillbirth occurred in patient exposed to Betaseron, and spontaneous abortions occurred in patients exposed to Rebif (2 of them) and Avonex.

Our patients had no relapses during pregnancy or breastfeeding. We restarted immunomodulatory treatment within 4 months after birth, except for one case who insisted to breastfeed for 6 months, at which time she made a relapse that increased her EDSS score by 1 point. The mean EDSS score in patients who remained pregnant was 2.1.

## 3. The Impact of the MS Treatment on Pregnancy

Since more and more therapeutic options have been released and implemented, we have performed a review of the impact that these drugs can have on the mother and the developing fetus. To achieve this, we reviewed information from the pregnancy registries about various therapies and the latest publications on the topic.

The interferons (Avonex, Betaseron, and Rebif) are all “Category C” drugs, meaning they caused some harm to fetuses in animal studies [4]. The Avonex Pregnancy Exposure Registry analyzed women with MS who were exposed to intramuscular (IM) IFNbeta-1a, approximately 1 week prior to conception or during the first trimester of pregnancy. Of the 306 outcomes, there were 272 live births, 28 spontaneous abortions, 5 induced abortions, and 1 stillbirth. The rate of spontaneous abortions (SAB) of 10.5% was comparable to the rate in the general population of 15%. Birth defects (spina bifida, Down syndrome, diaphragmatic hernia, duodenal atresia, hypospadias, club foot, trisomy 8, nuchal translucency, pyloric stenosis, and hydronephrosis) were reported in 17 infants and were consistent with those observed in the general population [5].

The Betaseron Pregnancy Registry was a follow-up study, with 86 live births, 2 stillbirths, and 11 SABs. The prevalence of SAB was 11.5%, with no significant difference from the 16% estimation for the general population. Stillbirths occurred in black women with both comorbidities and a history of prior SAB. There were 5 cases of birth defects (Down syndrome, hemangioma, polydactyly, ventricular septal defect, hip dysplasia, and patent foramen ovale), all exposed to interferon beta-1b during the first trimester of gestation, but the prevalence was not significantly different from the general population [6].

Other studies have demonstrated the association of changes in other parameters such as lower birth weight [7], shorter gestational period [8], or higher SAB rates [9] in interferon beta-exposed pregnancies.

Glatiramer acetate (Copaxone) is the only DMT with an FDA pregnancy “Category B” drug, meaning it did not cause harm to fetuses in animal studies. GA does not cross the placental barrier and it may be considered compatible with breastfeeding, because it is hard to believe that an amino acid polymer like GA could get entirely absorbed through an infant's gastrointestinal mucosa [10]. In a study on 44 women, only 7 patients discontinued GA, 9 remained on GA but discontinued when pregnant, and 28 remained on GA. The results were 28 normal children and 3 ongoing, one minor congenital anomaly, two pregnancies with Down syndrome who were terminated, one ectopic pregnancy, and 3 spontaneous abortions. These results suggest that GA may be safely continued during pregnancy [11].

There are studies that compared pregnancies under interferon-beta and under glatiramer acetate. The obstetric complications rates were similar for women who were exposed and who were not exposed to DMT. There has been one case of prematurity during the use of GA, but this was a patient that had three previous illegally provoked abortions. Another non-drug-related adverse event was a case of bone malformations in a mother exposed to GA [12]. No pattern of malformation was assigned to the first line MS drug (IFN-beta and GA) [13, 14]. Birth weight did not differ significantly from controls in pregnancies exposed to GA. There was a smaller difference between children whose mothers were or were not exposed to DMT and no significant difference between the heights of the children whose mothers had been exposed to GA or IFN [7, 8]. The relapse rate in mothers exposed in the first 8 weeks of pregnancy to DMT was significantly lower during pregnancy and after delivery and less EDSS progression was recorded in comparison with patients that were not exposed [12, 15].

Patients with a more aggressive disease may be treated with natalizumab (Tysabri) or fingolimod (Gilenya). Given abnormalities seen in animal studies, there have been observed abnormalities that advise stopping the medication 2 or 3 months before conception. Natalizumab is a Category C drug and a reduction in pregnancy rates was observed in guinea pigs. No adverse effects were reported on male fertility [16]. Natalizumab crosses the placenta in the second trimester and is secreted in small amounts in the human milk [17]. The results from the Tysabri Pregnancy Exposure Registry showed a spontaneous abortions rate of 11.2% in 23 pregnancies, with no significant differences when compared with the general population. In 35 women exposed to natalizumab during accidental pregnancies, 28 healthy neonates and one child with 6 fingers were born and five early miscarriages were recorded [18].

Fingolimod (pregnancy Category C) is teratogenic in rats, with congenital abnormalities reported [19, 20]. In the fingolimod clinical trial program, 34 pregnancies resulted in 13 healthy infants, 1 with a tibia malformation believed to be unrelated to treatment, 5 spontaneous abortions, 9 elective ones, and 6 ongoing pregnancies [21]. Fingolimod crosses the placenta and is secreted in breast milk. It is currently advised that patients with MS cease fingolimod for at least 2 months before getting pregnant. This is because fingolimod remains in the blood for at least 2 months after stopping the treatment.

Dimethyl fumarate (BG-12) is a pending approval pregnancy category. There was no teratogenicity or evidence of infertility found in rats and rabbits. Cell hyperplasia in the testis was observed in male rats at all doses, but with no effect on fertility. In females, there were no consequent effects on fertility [22]. In an older study on rats, a dosage of 508 mg/kg induced death, 356 mg/kg induced delay of ossification, and 178 mg/kg increased embryo absorption [23]. There were 34 pregnant subjects who received BG-12 with 65% live births compared with 64% in placebo-treated Subjects, 9% spontaneous abortion rate and no fetal abnormalities, consistent with the expected rate in the general population (12–22%). For the time being, it is still unknown if dimethyl fumarate crosses the placenta or is secreted in breast milk [24].

Mitoxantrone is a Category D drug that causes growth retardation and premature births in animals [25]. Patients should not become pregnant while taking mitoxantrone and they should wait at least 6 months after discontinuation [10]. Placental transfer of mitoxantrone is limited; it is secreted in milk and contraindicated when breastfeeding [4].

Teriflunomide is a Category X drug that causes teratogenicity in animals. It is contraindicated in pregnant women or with childbearing potential, who are not using reliable contraception. Teriflunomide crosses the placenta and is detected in rat milk, following a single oral dose [26].

Laquinimod is in the category of pending approval. Animal studies demonstrated fetal malformation in rats, but not in rabbits [27]. Women with childbearing potential are advised to use effective contraception and no breastfeeding is recommended [28].

Daclizumab, a monoclonal anti-CD25 antibody is a Category C drug, with no fetal malformation observed in monkeys, but with an increase in prenatal loss. Very low concentrations of daclizumab are secreted in the breast milk of lactating monkey. Recommendations are to use contraception and to avoid breastfeeding [29].

Short courses of steroids have been regarded as safe in pregnancy [30], in the second and third trimesters of pregnancy. Steroids cross the placental barrier and may increase the risk of cleft palate and low birth weight when used in the first trimester. Prednisone, prednisolone, and methylprednisolone can be administered with low levels of fetal exposure and may be preferred for use during pregnancy [31].

A promising treatment in the period around childbirth is intravenous immunoglobulins (IVIg). They appear to have no significant side effects on pregnancy and to reduce relapse rates [32, 33].

## 4. Breastfeeding

Breastfeeding should be encouraged, because there is no risk of disease transmission through breast milk. If the mother should receive a drug, it must be known that it is possible to be excreted in milk.

MS itself does not pose any obstacles to breastfeeding. Women who breastfed exclusively had significantly lower postpartum disease activity compared with women who did not breastfeed exclusively or did not breastfeed at all [15, 34]. A possible explanation might be that only exclusive breastfeeding on demand suppresses ovarian function, with high prolactin levels [35]. In experimental settings, high levels of prolactin were shown to promote remyelination [36]. Breastfeeding mothers are advised not to start DMT after birth, as there are no reliable data available on drug transfer into milk and the effects on newborns [37]. For the women who do not want to breastfeed, the recommendation is to start DMT as soon as possible after birth, because of the delayed onset of efficacy for IFN-beta and GA [38].

Fragoso et al. reported on 9 mothers who breastfed for a mean period of 3.6 months while taking GA. No significant ill effects were observed in those children during or after breastfeeding [39]. Hellwig and Gold followed 3 mothers taking GA and one taking IFN-beta during breastfeeding without any noticeable problems [14].

## 5. Difficulties during Pregnancy

During pregnancy, there can be apparent worsening of preexisting dysfunction or the occurrence of new events. Women who have walking impairments may find these getting worse during late pregnancy, as the patient becomes heavier and their center of gravity shifts. Excess weight can decrease motility already affected by the process of demyelination more or less extensive at the corticospinal fibers. Increased use of assistive devices to walk or the use of a wheelchair may be advisable at these times.

Preexisting dysfunctions may be of motor sphincter, digestive track, sensory activity, and fatigue and seem to be due to the state of pregnancy associated with weight gain. Bladder and bowel problems, which occur in all pregnant women, may be aggravated in women with MS who have preexisting urinary or bowel dysfunction. The presence of localized spinal demyelinating plaques associated with the uterus impact on thoracic-abdominopelvic cavity can generate or amplify urgency, constipation, and mild ventilatory dysfunction [40].

## 6. Difficulties at Birth

Labor and delivery are usually the same as in other women and no special management is needed. But in some cases, the obstetrician and the anesthetist must choose the safest option for the mother and fetus, taking into consideration the existing neurological dysfunction.

MS has no contraindications, neither for natural birth nor for caesarean section. Both epidurals and anesthesia for caesarean births are safe in women with MS. Neither breastfeeding nor epidural analgesia has got worse disability in the postpartum period [1]. Special consideration may have to be taken for the minority of people with MS who are severely disabled or have respiratory problems.

## 7. The Impact of Pregnancy on MS Activity

During pregnancy, the evolution of our patients has been positive, with a much lower relapse rate. These observations are confirmed by a 10-year follow-up study that found a lower relapse rate in women with pregnancies after disease onset, compared to those without pregnancies after MS onset [41]. A prospective five-year study compared the rate of progression in disability between childless women, women who had onset of MS after childbirth, and women who had onset before or during their pregnancy. The rates of disability, including wheelchair dependence, increased most rapidly in women with no children [42] and the risk of conversion to secondary progressive MS was lower [43].

There have been many studies examining the impact of pregnancy on MS. They all show that pregnancy appears to have a positive protective influence, with relapse rates going down, especially during the third trimester. The reasons are unknown, but it is believed that hormone fluctuations levels in pregnancy could have a role. The hormone involved in altering the immune system is estrogen. During pregnancy, estrogen levels rise and are the highest in the last trimester. This is considered to suppress the activity of the immune system, consequently decreasing the disease activity. After the birth, estrogen levels rapidly drop and the immune system returns to its usual function and MS resumes its work [44].

Another explanation for the association of pregnancy with spontaneous remission and the postpartum period with exacerbations arises from an immunologic point of view. The fetal placental unit secretes cytokines such as interleukin-10, resulting in an increased number of T helper 2 cells instead of T helper 1. The tolerance of the fetus by the mother may be explained through this mechanism. An inversion of this cytokine balance occurs at birth and it could be regarded as a graft-rejection process [45].

## 8. Disease Transmission and Fertility

It is known that multiple sclerosis is not directly inherited like other genetic diseases, and the patients are usually informed about the transmission incidence. The evidence shows that if the mother or father has MS, risk of getting the disease is 3–5% compared to the general population, where the probability is only 0.2%. If both parents have MS, the risk increases to 29.5%.

There are currently no genetic or prenatal tests, nor even tests on newborn, able to determine the probability of this circumstance. The risk in the general population is one out of every 800 children. In conclusion, although having a parent with MS increases the risk, the transmission incidence rate is still very small.

Having multiple sclerosis (MS) does not seem to affect fertility in male or female in any significant way. The standard immunomodulation agents for MS do not affect fertility itself. Mitoxantrone, an FDA-approved medication for progressive MS, may affect fertility in the same way that other chemotherapies do.

Although MS has not been shown to affect fertility, approximately 15% of all couples have trouble conceiving. There have been 3 studies focused on this issue. It seems that one type of the medications used, GnRH agonists, may increase the relapse rate. This effect is not seen with GnRH antagonists. This increase in relapse rates is present for at least 3 months following treatment [46]. Failure of in vitro fertilization (IVF) was also shown to be associated with an increased relapse [47]. Treatment with IFN and, particularly, GA appears safe in the perinatal period and indication is to continue treatment when having IVF.

## 9. Conclusion

Increasingly more women with multiple sclerosis want to have a baby. The decision to continue or discontinue the disease-modifying treatment belongs to the patient, together with her neurologist and her obstetrician. Patients must know all aspects of the adverse effects of therapy on themselves and on pregnancy and be aware of the benefits of continuing treatment during pregnancy.

The rate of relapse declines in pregnant women, especially in the third trimester, but before returning to the prepregnancy rate, the risk of relapse increases during the first three months postpartum.

Disease-modifying therapy has not proven a 100% safe administration throughout pregnancy or while breastfeeding, which is why we need a larger number of studies to assess the safety profile. According to statistics and studies having been conducted so far, GA seems to be the only available treatment that patients can trust in case pregnancy occurs under therapy and it can be used with certain safety.
